# Inhibition of UCHL1 by LDN-57444 attenuates Ang II–Induced atrial fibrillation in mice

**DOI:** 10.1038/s41440-019-0354-z

**Published:** 2019-11-07

**Authors:** Hai-Lian Bi, Yun-Long Zhang, Jie Yang, Qing Shu, Xiao-Lei Yang, Xiao Yan, Chen Chen, Zhi Li, Hui-Hua Li

**Affiliations:** 1 Department of Cardiology, Institute of Cardiovascular Diseases, First Affiliated Hospital of Dalian Medical University, Dalian, 116000 China; 2 Department of Nutrition and Food Hygiene, School of Public Health, Dalian Medical University, Dalian, 116044 China; 3 Affiliated Zhongshan Hospital of Dalian University, Dalian University, Dalian, 116000 China

**Keywords:** Angiotensin II, Atrial fibrillation, Deubiquitinase, Fibrosis, UCHL1

## Abstract

Atrial fibrillation (AF) is the most common human arrhythmia in clinical practice and may be promoted by atrial inflammation and fibrosis. Ubiquitination is an important posttranslational modification process that is reversed by deubiquitinating enzymes (DUBs). DUBs play critical roles in modulating the degradation, activity, trafficking, and recycling of substrates. However, less research has focused on the role of DUBs in AF. Here, we investigated the effect of ubiquitin C-terminal hydrolase 1 (UCHL1), an important DUB, on the development of AF induced by angiotensin II (Ang II). Male wild-type mice were treated with the UCHL1 inhibitor LDN57444 (LDN) at a dose of 40 μg/kg and infused with Ang II (2000 ng/kg/min) for 3 weeks. Our results showed that Ang II-infused wild-type (WT) mice had higher systolic blood pressure and an increased incidence and duration of AF. Conversely, this effect was attenuated in LDN-treated mice. Moreover, the administration of LDN significantly reduced Ang II-induced left atrial dilation, fibrosis, inflammatory cell infiltration, and reactive oxygen species (ROS) production. Mechanistically, LDN treatment inhibited the activation of multiple signaling pathways (the AKT, ERK1/2, HIF-1α, and TGF-β/smad2/3 pathways) and the expression of CX43 protein in atrial tissues compared with that in vehicle-treated control mice. Overall, our study identified UCHL1 as a novel regulator that contributes to Ang II-induced AF and suggests that the administration of LDN may represent a potential therapeutic approach for treating hypertensive AF.

## Introduction

Atrial fibrillation (AF), the most common clinical arrhythmia worldwide, has been well characterized, and it is associated with serious cardiovascular diseases and an increased risk of stroke and death. Although the pathophysiological mechanism of AF is not fully understood, atrial electrical and structural remodeling are the main factors of the persistence and progression of AF [1, 2]. Atrial fibrosis is the hallmark of atrial structural remodeling, which is a common feature of clinical AF [3]. Increasing evidence has demonstrated that the renin–angiotensin system (RAS) plays a key role in the initiation and development of AF. AngII (angiotensin II) exerts its biological functions via the activation of angiotensin type 1 receptor (AT1R) [4]. The activation of AT1R stimulates several downstream mediators, including AKT/ERK, TGF-β/Smad, MAP kinases, NADPH oxidase, NF-kB, and hypoxia inducible factor-1alpha (HIF-1α), which are involved in vasoconstriction, fibrosis, inflammation, oxidative stress, and ion channel abnormalities [1, 5–7], Ang II-mediated inflammation and oxidative stress are thought to promote atrial fibrosis in AF [2, 8, 9]. Thus, the inhibition of the AT1R-mediated activation of signaling is critical for blocking AF development.

Ubiquitination is a dynamic posttranslational modification that is naturally mediated by ubiquitin-activating (E1), ubiquitin-conjugating (E2), and ubiquitin ligase (E3) enzymes. This modification can be reversed by deubiquitinases (DUBs, also called deubiquitinating enzymes), which can regulate the degradation, trafficking, and activity of substrates [10]. Ubiquitin C-terminal hydrolase 1 (UCHL1), also called protein gene product 9.5 (PGP9.5), is an important member of the ubiquitin carboxy-terminal hydrolase (UCH) family of DUBs and catalyzes the hydrolysis of C-terminal ubiquityl esters and amides. Increasing data has indicated that UCHL1 plays an important role in proliferation, neoplasm metastasis, oxidative stress and immune responses, and it has been implicated in the development of neurodegenerative diseases and cancer [11–14]. A study suggested that UCHL1 influences skeletal muscle development and function. The deletion of UCHL1 in mice results in abnormal shuffling movements, hind-limb paralysis, and early death [15]. Recently, UCHL1 expression was shown to be highly upregulated in cardiomyocytes after myocardial infarction and to be associated with increased ubiquitin expression [16]. However, little is known about the role of UCHL1 in regulating AF and atrial remodeling.

In this study, we examined the effect of UCHL1 inhibition by LDN, a specific inhibitor, on the development of AF in a murine model of Ang II-induced AF. Our results showed that Ang II infusion significantly upregulated UCHL1 expression at both the mRNA and protein levels in the atria. The administration of LDN to mice reduced the Ang II-induced elevation of blood pressure, the inducibility and duration of AF, left atrial dilation, fibrosis, inflammation, and oxidative stress. This protective effect was associated with the inhibition of multiple signaling pathways (the AKT, ERK1/2, HIF-1α, TGF-β/Smad2/3, and CX43 pathways). Thus, these results suggest that the inhibition of UCHL1 attenuates Ang II-induced AF. The UCHL1 inhibitor LDN may be a potential therapeutic drug for the treatment of hypertensive AF.

## Materials and methods

### Animals and treatment

Eight-week-old male C57BL/6 mice (the Jackson Laboratory, Bar Harbor, ME, USA) were infused with saline or Ang II (2000 ng/kg/min) (A107852; Aladdin, Shanghai, CHN) using osmotic mini-pumps (Alzet model 1004; Durect, Cupertino, CA, USA) for 3 weeks as described previously [17]. The UCHL1 inhibitor LDN-57444 (40 μg/kg) (MCE, Burlington, NJ, USA) or vehicle (DMSO) (Sigma-Aldrich, St. Louis, MO, USA) was administered intraperitoneally one time per day for 3 weeks beginning one day before Ang II infusion. This entire study was approved by the Animal Care and Use Committee of Dalian Medical University and conformed to the Guide for the Care and Use of Laboratory Animals published by the U.S. National Institutes of Health (NIH Publication No. 85-23, revised 1996).

### Blood pressure measurement

The blood pressure of the mice was measured one day before LDN was administered and monitored every other day after Ang II infusion with a tail-cuff system (BP-2010, Softron, Tokyo, JPN), as described previously [18, 19].

### Arrhythmia inducibility and duration

After 3 weeks of treatment, the mice were anesthetized by an intraperitoneal injection of 2.5% tribromoethanol (0.02 mL/g; Sigma-Aldrich, St. Louis, MO, USA) [20]. At the end of Ang II infusion, intracardiac pacing was performed in mice through the insertion of an eight-electrode catheter (1.1F, octapolar EP catheter, Scisense, London, Ontario, Canada) via the jugular vein and its advancement into the right atrium and ventricle [20]. The inducibility of AF was measured by applying a 5-s burst using an automated stimulator as described previously and recorded with a computer-based data acquisition system (GY6328B; Henan Huanan Medical Science and Technology, Co., Ltd., Zhengzhou, HA, CHN) [4, 20]. A series of bursts were repeated three times after stabilization for 5 min. The duration of AF was defined as the time interval between a rapid and irregular atrial rhythm following the onset of AF and the onset of the first normal sinus beat.

### Histopathologic examinations

The hearts were removed and fixed in 4% paraformaldehyde (Solarbio, Beijing, CHN) for 24–48 h and then embedded in paraffin. The samples were sectioned into 5-μm sections and stained with Masson’s trichrome (Sigma-Aldrich, St Louis, MO, USA) following the manufacturer’s instructions as previously described [18]. Immunohistochemical staining was performed with anti-UCHL1, α-SMA (α-smooth muscle actin) (ab7817; Abcam, Bristol, UK), F4/80 (ab6640; Abcam, Bristol, UK) antibodies [18]. Cryosections were prepared and stained with dihydroethidine (DHE; Sigma-Aldrich, St. Louis, MO, USA) as described [18]. Images of ten random fields per sample were taken at ×100 or ×200 magnification (Nikon, Tokyo, JPN) and were analyzed by ImageJ (NIH, Bethesda, MD, USA).

### Quantitative real-time PCR (qPCR) analysis

Total RNA was isolated from LA tissues with TRIzol (Invitrogen) and reverse transcribed using a reverse transcription kit (RR047A; Takara, Tokyo, JPN) to synthesize first-strand cDNA [18]. qPCR amplification was performed using an iCycler IQ system (Bio-Rad, Plano, TX, USA). Primers used in the qPCR analysis are shown in Supplementary Table 1 (Sangon Biotech, Shanghai, CHN). The relative mRNA levels were normalized to those of the endogenous control (GAPDH).

### Western blot analysis

Snap-frozen left atrial tissues were prepared and subjected to sonication in ice-cold RIPA buffer containing inhibitors and PMSF (100 mM; Solarbio, Beijing, CHN). Proteins were separated by electrophoresis on SDS-PAGE gels and blotted onto PVDF membranes (Millipore, Billerica, MA, USA). The appropriate antibodies, including UCHL1 (14730-1-AP; Proteintech, Wuhan, HB, CHN), AKT (#9272; Cell Signaling Technology, Danvers, MA, USA), p-AKT (Ser473) (#4060; Cell Signaling Technology, Danvers, MA, USA), ERK1/2(#9102; Cell Signaling Technology, Danvers, MA, USA), p-ERK1/2 (Thr202/Tyr204) (#9101; Cell Signaling Technology, Danvers, MA, USA), TGF-β (#3711; Cell Signaling Technology, Danvers, MA, USA), Smad2 (#5339; Cell Signaling Technology, Danvers, MA, USA), p-Smad2/3(#8828; Cell Signaling Technology, Danvers, MA, USA), CX43 (ab11370; Abcam, Bristol, UK) and α-tubulin (11224-1-AP; Proteintech, Wuhan, HB, CHN) antibodies, were used [18]. The blots were developed by using the enhanced chemiluminescent system (ECL Plus, Thermo Fisher, Waltham, MA, USA), and signal acquisition was performed using the FluorChem M system (ProteinSimple, San Jose, CA, USA). The intensities of the proteins were measured by ImageJ software and were normalized to the intensity of endogenous tubulin.

### Statistical analysis

Statistical calculations were performed using SPSS 16.0 (SPSS, Chicago, IL, USA). All results are expressed as the mean ± standard error of the mean (SEM). First, a normality test was performed. If the data were normally distributed, Student’s *t* test was used to test the difference between the two groups. If the data were not normally distributed, the Mann–Whitney *U* test was used to test the difference between the two groups. *P* < 0.05 was considered statistically significant.

## Results

### Ang II infusion upregulates UCHL1 expression in the Atria

**Fig. 1:**
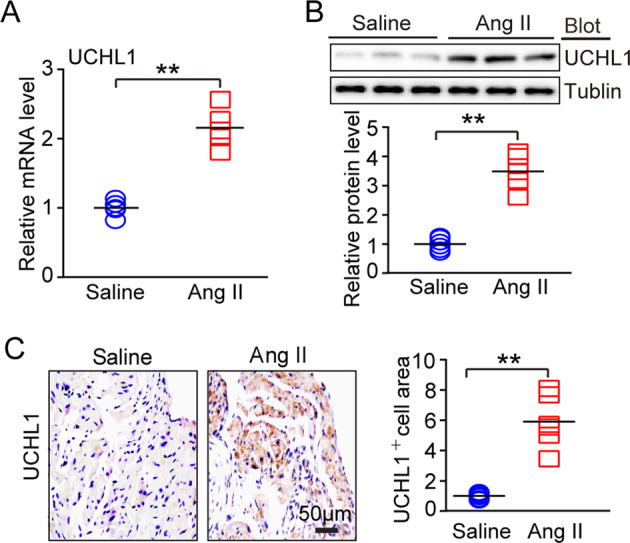
Ang II infusion upregulates UCHL1 expression in the hearts. **a** Wild-type (WT) mice were infused with saline or Ang II (2000 ng/kg/min) for 3 weeks. UCHL1 mRNA expression in the atrial tissues was detected by qPCR analysis. **b** UCHL1 protein levels in atrial tissues were detected by immunoblotting analysis (upper) and quantified (lower). **c** Atrial sections were stained with an anti-UCHL1 antibody by immunohistochemistry (left). Scale bar = 50 μm. The quantification of the UCHL1-positive area (right). ^*^P *<* 0.05, ^**^P < 0.01 vs saline-treated WT mice

To investigate whether UCHL1 activity is involved in regulating AF, we first tested the expression of UCHL1 in atrial tissues. The mRNA and protein levels of UCHL1 were significantly upregulated in Ang II-infused atrial tissues after 3 weeks of infusion (2000 ng/kg/min) compared with saline-infused tissues (Fig. 1a, b). Furthermore, the increase in UCHL1 expression in Ang II-infused atrial tissues was confirmed by immunohistochemical staining (Fig. 1c). These data suggest that UCHL1 may play a role in Ang II-induced AF.

### Administration of LDN inhibits Ang II-induced systolic blood pressure and left atrial (LA) dilation

**Fig. 2:**
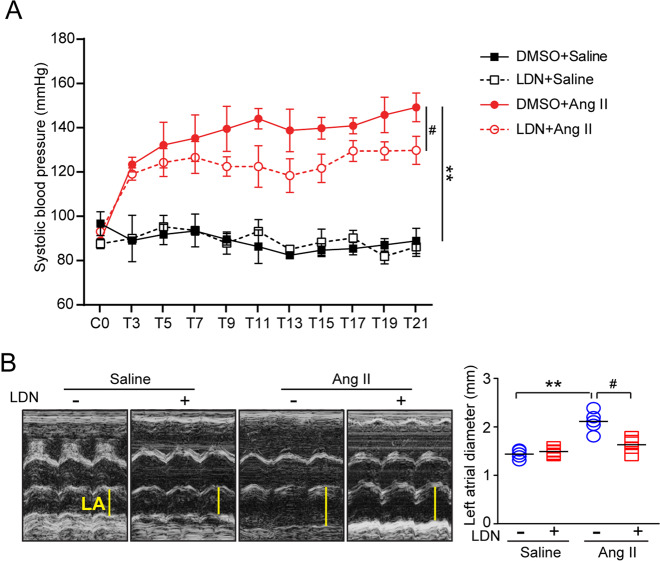
Administration of LDN reduces pressure blood pressure elevation and improves atrial dysfunction after Ang II infusion. Male wild-type (WT) mice were infused with Ang II and injected intraperitoneally with LDN for 3 weeks. **a** Systolic blood pressure (SBP) was measured by a mouse tail-cuff system before and after Ang II infusion. **b** Representative echocardiographs of LA dilation in wild-type mice treated with LDN or DMSO (control) after 3 weeks of saline or Ang II infusion. ^*^P *<* 0.05, ^**^P < 0.01 versus saline control; ^#^*P* < 0.05 versus Ang II alone

We next determined whether the inhibition of UCHL1 activity regulates systolic blood pressure (SBP) and atrial structure remodeling. We established an AF mouse model through the infusion of a high dose of Ang II and administered the UCHL1 inhibitor LDN. After 3 weeks of Ang II treatment, the SBP was elevated in the vehicle-treated mice compared with the saline-treated mice. However, this effect was significantly reduced in the LDN-treated mice (Fig. 2a). Since atrial structural remodeling is a major risk factor for AF [4], we then examined the effect of LDN on atrial dilation in vivo. Echocardiography revealed that the Ang II-induced dilation of the left atrium (LA) observed in vehicle-treated WT mice was also attenuated in LDN-treated mice (Fig. 2b).

### Administration of LDN Attenuates Ang II-induced AF

**Fig. 3:**
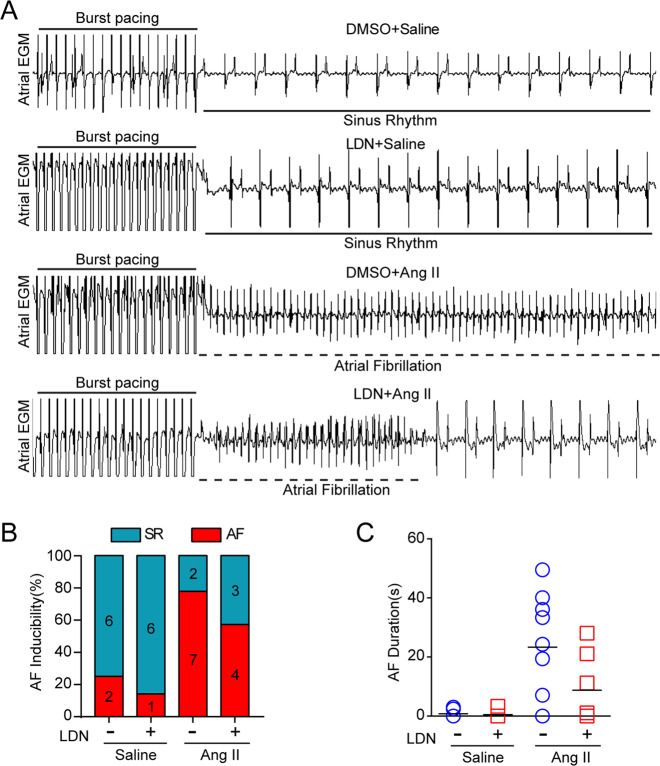
Administration of LDN attenuates Ang II-induced AF. **a** Representative atrial electrogram recordings of DMSO-treated and LDN-treated mice infused with saline or Ang II for 21 d. Burst pacing is indicated by the solid lines, and the dashed lines indicates AF. **b** The percentage of mice in which AF was successfully achieved in each group (bottom left; *n* = 7–9 mice per group). **c** Total AF duration for each DMSO-treated and LDN-treated mouse infused with saline or Ang II, as determined by burst pacing

To determine the role of LDN in regulating AF development, the inducibility and duration of AF were examined in Ang II-infused mice treated with or without LDN. The inducibility of AF was appreciably increased in the Ang II-treated mice compared with the saline-treated mice (77.8% versus 25.0%). Although LDN treatment reduced Ang II-triggered AF inducibility (57.1% versus 77.8%), no significant difference was observed between the LDN-treated mice and the vehicle (DMSO)-treated mice (Fig. 3a, b). Interestingly, the total duration of AF was substantially reduced in the LDN-treated mice compared with the vehicle-treated mice after Ang II infusion (Fig. 3c). There was no significant difference between the two groups treated with saline (Figs. 3a through c). The heart rate was not significantly changed between the vehicle-treated and LDN-treated mice after saline or Ang II infusion.

### Administration of LDN attenuates Ang II-induced fibrosis

**Fig. 4:**
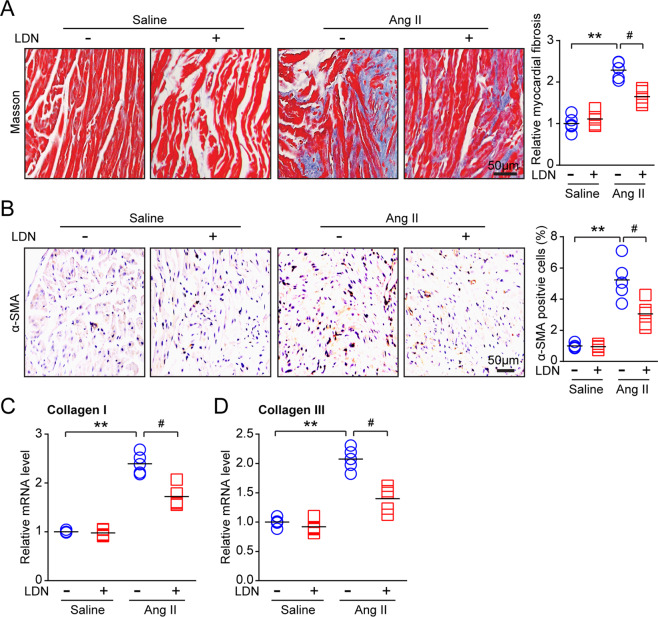
Treatment with LDN suppresses Ang II-induced atrial fibrosis. Male wild-type (WT) mice were infused with Ang II and injected intraperitoneally with LDN (40 μg/kg, one time per day) for 21 d. **a** Representative images of Masson trichrome staining for atrial fibrosis (left). The quantification of the fibrotic area (right; *n* = 5 mice per group). **b** Representative images of α-SMA (a fibrosis marker) immunohistochemistry (left). Then quantification of α-SMA-positive cells (right; *n* = 5 mice per group). **c**, **d** qPCR analyses of collagen I and collagen III levels in atrial tissues. *n* represents the number of animals. ^*^P *<* 0.05, ^**^P < 0.01 versus saline control; ^#^*P* < 0.05 versus Ang II alone

We then examined the role of LDN on atrial fibrosis, a hallmark of atrial remodeling. Ang II treatment, compared with saline treatment, significantly increased the atrial fibrotic area, whereas LDN treatment remarkably attenuated this effect (Fig. 4a). Moreover, the increase in the number of α-SMA-positive myofibroblasts induced by Ang II infusion observed in the vehicle (DMSO)-treated WT mice was markedly attenuated in the LDN-treated mice (Fig. 4b). In addition, the increased mRNA expression of fibrotic markers (collagen I and III) induced by Ang II was also reduced in the LDN-treated mice (Fig. 4c, d). Together, these data suggest that the administration of LDN inhibits Ang II-induced hypertension, left atrial dilation and fibrosis.

### Administration of LDN attenuates Ang II–induced inflammation and oxidative stress

**Fig. 5:**
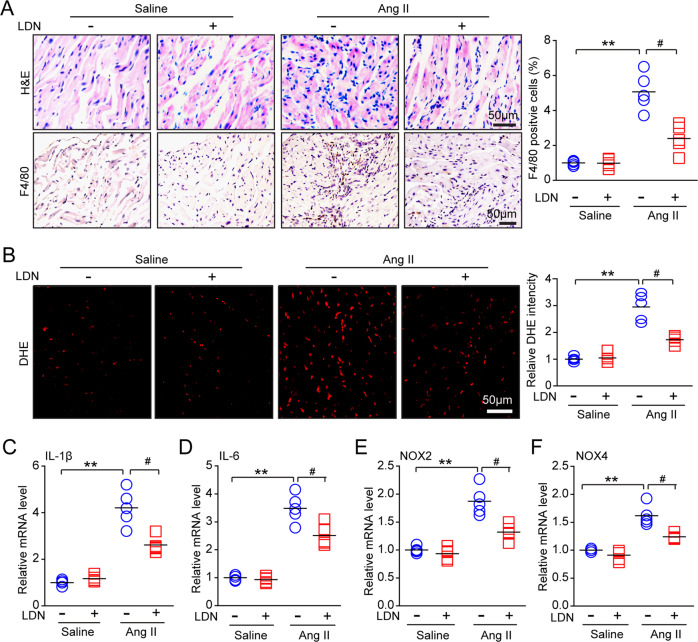
Administration of LDN reduces Ang II-induced atrial inflammation. **a** Representative hematoxylin-eosin (H&E) staining (top) and F4/80 (macrophage marker) immunohistochemistry (bottom) in the atrial tissues of DMSO-treated and LDN-treated mice after infusion with saline or Ang II for 21 d (*n* = 5 mice per group). The quantification of F4/80-positive cells (right; *n* = 5 mice per group). **b** Dihydroethidium (DHE) staining of atrial tissues from DMSO-treated and LDN-treated mice after infusion with saline or Ang II for 21 d (left). The quantification of fold changes in DHE intensity (right; *n* = 5 mice per group). **c**–**f** qPCR analyses of proinflammatory cytokine (IL-1β and IL-6) and NADPH oxidase subunit (NOX2 and NOX4) levels in atrial tissues (*n* = 5). n represents the number of animals. ^*^P *<* 0.05, ^**^P < 0.01^,^ or ^#^*P* < 0.05 versus saline-treated WT mice or Ang II-treated WT mice

It has been reported that inflammation and oxidative stress play a critical role in regulating atrial fibrosis [21]. We further assessed whether LDN influences the inflammatory response and reactive oxygen species (ROS) production in the atria. Ang II infusion resulted in a marked increase in inflammatory cell infiltration, including that of F4/80-positive macrophages, in the vehicle-treated mice, but this increase was attenuated in the LDN-treated mice (Fig. 5a). Moreover, DHE staining revealed that the Ang II-induced increase in superoxide production observed in WT mice was decreased in LDN-treated mice (Fig. 5b). Accordingly, the mRNA levels of IL-1β and IL-6 (proinflammatory cytokines) and NOX2 and NOX4 (NADPH oxidase subunits) were lower in atrial tissue from the LDN-treated mice than in tissue from the DMSO-treated mice with Ang II infusion (Fig. 5c–f). There was no significant difference in these parameters between the two groups treated with saline (Fig. 5a–f).

### Administration of LDN reduces multiple signaling pathways

**Fig. 6:**
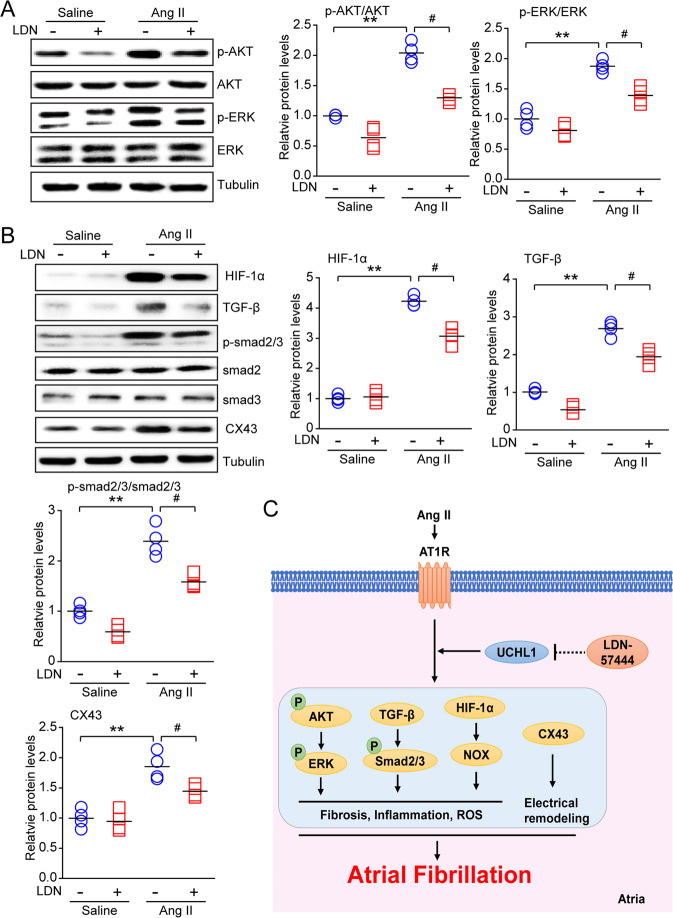
Treatment of LDN suppresses Ang II-induced oxidative stress, inflammation and downstream mediators in mice. **a** Immunoblotting analyses of the profibrotic signaling molecules p-AKT, AKT, p-ERK1/2, ERK1/2, and tubulin in the heart. The quantification of the relative protein levels (*n* = 4). **b** Immunoblotting analyses of fibrosis, oxidative stress and the inflammatory mediators HIF-1α, TGF-β1, p-Smad2/3, Smad2, Smad3, and CX43 in the heart. The quantification of the relative protein levels (*n* = 4). α-Tubulin was used as an internal control. n represents the number of animals (*n* = 4). ^*^P *<* 0.05, ^**^P < 0.01 versus saline control; ^#^*P* < 0.05 versus Ang II alone. **c** A working model of the effect of LDN administration on Ang II-induced AF. Treatment with LDN suppresses Ang II-induced oxidative stress and inflammatory mediators (AKT, ERK1/2, HIF-1α, and TGF-β/Smad2/3) in mice

Ang II triggers multiple signaling pathways, such as AKT/mTOR, HIF-1α, TGF-β/ Smad2/3, and NADPH oxidase, which are involved in inflammation and fibrosis formation [20, 22]. To study the molecular mechanism by which LDN protects against Ang II-induced AF, we investigated several signaling pathways associated with atrial fibrosis and inflammation. The administration of LDN, compared with vehicle, attenuated Ang II-induced increases in p-AKT, p-ERK, HIF-1α, TGF-β1, and p-Smad2/3 in the atria (Fig. 6a, b). In addition, the expression of CX43 was markedly attenuated in the LDN-treated mice compared with the vehicle (DMSO)-treated mice after Ang II treatment (Fig. 6c).

## Discussion

This study is the first to confirm that UCHL1 expression is significantly upregulated in Ang II-infused atrial tissues. The inhibition of UCHL1 by LDN (a specific inhibitor) significantly reduced the Ang II-induced increase in blood pressure, atrial fibrillation, fibrosis, inflammation, and oxidative stress. These beneficial actions were possibly associated with the inactivation of multiple signaling mediators (AKT, ERK1/2, HIF-1α, and TGF-β/Smad2/3) in atrial tissues (Fig. 6). The working model is shown in Fig. 6c. In summary, our results indicate that UCHL1 plays an important role in Ang II-induced AF and may be a novel target for the treatment of hypertensive AF.

UCHL1 was originally identified as a neuron-specific protein highly expressed in the nervous system, and its mutations are associated with neurodegenerative diseases, including Parkinson’s disease and Alzheimer’s disease [11, 12]. Previous studies have shown that UCHL1 is expressed at lower levels in healthy tissues, but it is highly induced in several forms of cancer and lung tumor cell lines [23]. UCHL1 is also upregulated in denervated skeletal muscles and pulmonary artery constriction (PAC)-treated right ventricular and infarcted heart tissues [16, 24, 25]. Moreover, UCHL1 has been implicated in regulating apoptosis of β cells and diabetes progression in hIAPP-Tg mice [26, 27]. In this study, our results showed that UCHL1 expression was also increased in the atria after Ang II infusion (Fig. 1), indicating that the upregulation of UCHL1 expression may influence AF susceptibility. Indeed, we demonstrated for the first time that the inhibition of UCHL1 activity by the inhibitor LDN attenuates Ang II-induced AF and atrial remodeling.

Ang II is involved in regulating various cardiovascular diseases, such as hypertension, cardiac hypertrophic remodeling, heart failure, and AF [28]. The activation of AT1R by Ang II triggers multiple signaling pathways, including the PI3K/AKT, MAPKs, NADPH oxidase, and TGF-β/Smad pathways, which stimulate atrial fibrosis, inflammation, and oxidative stress, leading to AF inducibility [4, 20]. Both the TGF-β/Smad and Hif-1α pathways have been demonstrated to be key signaling pathways in Ang II-induced myocardial fibrosis [1]. HIF consists of an α-subunit and a β-subunit. HIF-1α is a critical transcription factor that is closely related to hypoxia, and increased NOX expression results in ROS production [29]. TGF-β1 also induces CX43 expression via the Smad and ERK1/2 signaling pathways [30], which are involved in Ang II-stimulated AF [28]. Interestingly, these proinflammatory and profibrotic pathways are regulated by AKT, which activates NF-kB, NOX, and TGF-β/Smad signaling [20, 31]. Notably, recent data have demonstrated that UCHL1 can interact with AKT and lead to the activation of AKT in MCF-7 cells [32]. Whether the inhibition of UCHL1 blocks AKT and multiple downstream mediators remains unclear. LDN is a potent reversible, competitive and active site-directed inhibitor of UCHL1. The administration of LDN aggravates mossy fiber sprouting and induces LC3 puncta formation and autophagy in the HeLa cell line and apoptosis through the activation of endoplasmic reticulum stress (ERS) [33, 34]. Here, our results demonstrated that the treatment of mice with LDN significantly inhibited the Ang II-induced activation of AKT, ERK1/2, HIF-1α, TGF-β/Smad2/3, IL-1, IL-6, and NOX, thereby leading to the improvement of atrial remodeling and AF (Figs. 4–6**)**. Thus, the results provide novel insights into the mechanisms of Ang II-induced AF as well as into the protective effect of UCHL1 against AF. This effect is consistent with previous findings that LDN inhibits the activation of ERK and AKT in human neuroblastoma cell lines [35], suggesting that UCHL1 regulates these signaling pathways in different cell types.

Notably, although the reduction in blood pressure induced by LDN in Ang II-infused mice was significant, the SBP was still higher than that in saline-infused controls (Fig. 2a), suggesting that the administration of LDN partially reduced hypertension. Furthermore, LDN markedly attenuated Ang II-induced left atrial remodeling, inflammation and oxidative stress as well as the activation of multiple signals (AKT, ERK1/2, HIF-1α, TGF-β/smad2/3, and CX43) in the atria, suggesting that LDN inhibited AF possibly through blood pressure-dependent and blood pressure-independent manners. How hypertension is involved in the LDN-mediated beneficial effect against AF and whether low-dose LDN has the same effect on AF need to be tested in a pressure overload-induced AF model in the future.

In conclusion, this study clarifies a new role for the UCHL1 inhibitor LDN in Ang II-induced AF and the possible mechanism. These data suggest that LDN may have potential clinical applications for the treatment of hypertensive AF and related heart diseases. In the future, it is necessary to further investigate how hypertrophic stimulation upregulates the expression of UCHL1 in atrial tissues and to assess the role of LDN in other animal models.

## Supplementary information

Supplementary Table 1
