# Supplementary material for: Inhibition of UCHL1 by LDN-57444 attenuates Ang II–Induced atrial fibrillation in mice
Source: Hypertens Res. 2019 Nov 7;43(3):168–77. doi: 10.1038/s41440-019-0354-z (PMC8075865; doi:10.1038/s41440-019-0354-z)
Supplement: Supplementary file 1 — Supplementary Table 1 [file 41440_2019_354_MOESM1_ESM.docx]

Supplementary Table 1. Primers used for quantitative real-time PCR analysis

| Gene | Forward Primer | Reverse Primer |
| --- | --- | --- |
| UCHL1 | 5’- GCCCAGCATGAAAACTTCAG-3’ | 5’- TTTGCCTCTTCAACAGGGGA-3’ |
| Collagen I | 5’-AGTCGATGGCTGCTCCAAAA-3’ | 5’-AGCACCACCAATGTCCAGAG-3’ |
| Collagen III  IL-1β  IL-6  NOX2  NOX4 | 5’-TCCTGGTGGTCCTGGTACTG-3’  5’-CTTCCCCAGGGCATGTTAAG-3’  5’-TTCCATCCAGTTGCCTTCTTG-3’  5’-CTTCTTGGGTCAGCACTGGC-3’  5’-CTTGGTGAATGCCCTCAACT-3’ | 5’-AGGAGAACCACTGTTGCCTG-3’  5’-ACCCTGAGCGACCTGTCTTG-3’  5’-TTGGGAGTGGTATCCTCTGTGA-3’  5’-GCAGCAAGATCAGCATGCAG-3’  5’-TTCTGGGATCCTCATTCTGG-3’ |
| GAPDH | 5’-GGTTGTCTCCTGCGACTTCA-3’ | 5’-GGTGGTCCAGGGTTTCTTACTC-3’ |
